# Assessment of Mean Platelet Volume as an Inflammatory Marker in Chronic Obstructive Pulmonary Disease Exacerbations

**DOI:** 10.7759/cureus.89984

**Published:** 2025-08-13

**Authors:** Mohamed E Ahmed, Fatmaalzahraa S Abdalrazik, Azza H El-sayed

**Affiliations:** 1 Chest Diseases Department, Faculty of Medicine, Helwan University, Cairo, EGY

**Keywords:** airway abnormalities, chronic obstructive pulmonary disease exacerbation, full blood count, inflammatory marker, mean platelet volume

## Abstract

Background: Chronic obstructive pulmonary disease (COPD) exacerbations significantly affect patients by worsening their health status, decreasing physical activity, and accelerating lung function decline. Mean platelet volume (MPV) serves as platelet function critical marker in inflammatory diseases, including COPD, where inflammation is pivotal. Recent studies evaluating this issue have yielded limited and controversial results. This work aimed to evaluate MPV as an inflammatory marker during COPD exacerbations.

Methods: This prospective study included 68 COPD patients diagnosed based on the Global Initiative for Chronic Obstructive Lung Disease (GOLD) guidelines. Cases were allocated equally into stable COPD and exacerbated COPD groups.

Results: Initial and follow-up MPV were significantly decreased among the exacerbation group in comparison to the stable group. A significant direct correlation was found between initial and follow-up MPV levels, while an inverse relationship was observed between MPV and total leukocyte count (TLC) in the exacerbation group.

Conclusion: MPV is a promising inflammatory marker in COPD exacerbations, demonstrating significant decreases during acute episodes. Its correlation with systemic inflammation supports its potential as a negative acute-phase reactant, warranting further clinical validation.

## Introduction

Chronic obstructive pulmonary disease (COPD) is a diverse pulmonary disorder marked by enduring respiratory signs, including dyspnea, cough, and sputum production. This disorder is caused by airway abnormalities, such as emphysema, which lead to prolonged and often worsening obstructions to the flow of air through the lungs. Spirometry is the most widely utilized method for assessing pulmonary function, primarily due to its accessibility and ease of use. It is also employed to evaluate the limitations of airflow [1].

COPD exacerbation is characterized by a worsening of dyspnea and/or cough and sputum production within a period of 14 days. This is typically associated with an increase in systemic and local inflammation caused by airway infections, pollutants, or other lung irritants. Patients are substantially affected by COPD exacerbations, which result in a rapid decline in lung function, decreased physical activity, and impaired health status [2].

Platelets are recognized for their significant involvement in both inflammation and thrombosis. Mean platelet volume (MPV) quantifies platelet size and correlates with platelet activity [3]. MPV serves as an important indicator of platelet production rates and their activation [4]. Moreover, in inflammatory diseases, MPV acts as a marker of platelet function. Given the significant role of inflammation in COPD, it is anticipated that alterations in platelet activity and, consequently, MPV will occur [5].

MPV is derived from a standard complete blood count test. Prior research has demonstrated inverse correlations between disease activity and MPV in conditions such as rheumatoid arthritis, inflammatory bowel disease, and ankylosing spondylitis [6]. Multiple studies indicate disturbances in MPV levels among individuals with COPD [7]. However, the relationship between MPV and COPD exacerbation remains insufficiently assessed. Recent studies evaluating this issue have yielded limited and controversial results [8].

Multiple researchers have reported variations in MPV among COPD patients compared to control groups [9]. However, other investigators have found that the variance in MPV among COPD cases was not statistically validated [10]. This study aimed to evaluate the role of MPV as an inflammatory marker in COPD exacerbations.

## Materials and methods

Study design and setting

This prospective investigation was conducted on 68 COPD cases diagnosed according to the Global Initiative for Chronic Obstructive Lung Disease (GOLD) 2023 criteria [11]. These patients attended the pulmonary outpatient clinics at Badr University Hospital in Cairo, Egypt, between January 2024 and January 2025.

Inclusion criteria for stable COPD patients required evidence of chronic, progressive, and partially irreversible airflow limitation, a history of smoking, and confirmation through spirometry, with a minimum of four weeks since their last exacerbation. Patients were included in the exacerbation group if they experienced a sudden decline in respiratory health, characterized by worsening symptoms such as shortness of breath, coughing, and increased sputum production (GOLD, 2023). Exclusion criteria included individuals under 18 or over 70 years of age, non-smokers, those with chronic inflammatory diseases, patients on anticoagulant or antiplatelet therapy, and those with hematological, rheumatological, liver, or renal diseases. Individuals who declined participation were also excluded.

The patients were allocated into two equal groups: Group A, composed of stable COPD patients serving as the control group, and Group B, composed of patients diagnosed with exacerbated COPD. All participants underwent medical history taking, full clinical examinations, spirometry pulmonary function tests, and laboratory investigations (including complete blood count (CBC), assessing white blood cells, red blood cells, platelet count, and MPV).

The spirometry pulmonary function test is a physiological test that measures the ability to inhale and exhale air relative to time. It was employed as the diagnostic instrument to evaluate airflow obstruction and ascertain the severity of the disease. The main results of spirometry include forced vital capacity (FVC), forced expiratory volume at one second (FEV1), and the FEV1/FVC ratio. The procedure involves three phases: (i) maximal inspiration; (ii) "blast" exhalation; and (iii) continued complete exhalation to the end of the test. The procedure is usually performed in a standard sitting position; however, spirometry measurement in the supine position may be indicated for certain neuromuscular disorders. Normal findings of spirometry include an FEV1/FVC ratio greater than 0.70, with both FEV1 and FVC above 80% of the predicted value. The severity of the disease can be classified by spirometry as mild (with a predicted FEV1 of at least 80%), moderate (predicted FEV1 between 50 and 80%), severe (FEV1 30-49%), and very severe (predicted FEV1 less than 30%).

MPV was measured as part of the CBC and serves as an indicator of platelet size. In various conditions, including myocardial infarction, pre-eclampsia, unstable angina, and systemic inflammatory diseases such as Crohn's disease and ulcerative colitis, MPV is an extensively used marker of platelet function that reflects inflammatory burden and disease activity. Nevertheless, clinicians often fail to acknowledge its importance [12].

Statistical analysis 

Data were gathered, tabulated, and statistically analyzed using IBM SPSS Statistics (IBM Corp., Armonk, USA). Parametric data were presented as minimum, maximum, and mean ± SD. We used the unpaired t-test (t) to compare the two groups. The paired t-test was conducted to analyze the differences between two related variables (pre- and post-measurements) within the same group. Pearson correlation (r) was used to measure the relationship between two parametric variables. Non-parametric data were presented as numbers and percentages. The chi-square (χ^2^) test was used to compare the two groups. Two-tailed p-values > 0.05 were regarded as statistically insignificant, whereas p-values < 0.05 were considered statistically significant.

Ethical considerations

Prior to participation in the study, patients provided informed consent after receiving a clear explanation of the study's purpose, scope, and potential consequences. The study was approved by the Research Ethics Committee of the Faculty of Medicine at Helwan University, Cairo, Egypt (approval number: 5-2024).

## Results

All cases

Table 1 shows that the age ranged from 41 to 70 years, with a mean value (± SD) of 53.77 (± 7.74) years. Regarding gender, 52 (76.47%) patients were male and 16 (23.52%) patients were female. Body mass index (BMI) ranged from 17.5 to 29.1 kg/m² with a mean value (± SD) of 22.42 (± 2.81) kg/m².

**Table 1:** Sociodemographic data of all studied cases BMI: Body mass index

Variable		All cases (N = 68)
Age (years)	Min-Max	41-70
	Mean ± SD	53.77 ± 7.74
Gender	Male, n (%)	52 (76.47%)
	Female, n (%)	16 (23.52%)
BMI (kg/m^2^)	Min-Max	17.5-29.1
	Mean ± SD	22.42 ± 2.81

Table 2 shows that FEV1/FVC of the studied cases ranged from 0.34 to 0.69, with a mean (± SD) of 0.6 (± 0.08). FEV1% ranged from 21 to 97, with a mean (± SD) of 57.94 (± 22.42). The initial MPV ranged from 6.8 to 12.3 fL, with a mean (± SD) of 9.37 (± 1.46) fL; meanwhile, the follow-up MPV after one month ranged from 7.1 to 12.1 fL, with a mean (± SD) of 9.4 (± 1.22) fL. Total leukocyte count (TLC) of the studied cases ranged from 4.7 to 15.9, with a mean (± SD) of 9.29 (± 2.92). The platelet count of the studied cases ranged from 146 to 504, with a mean (± SD) of 300.5 (± 93.38).

**Table 2:** Spirometry and CBC analysis of all studied cases FEV1: Forced expiratory volume at one second; FVC: Forced vital capacity; MPV: Mean platelet volume; TLC: Total leukocyte count; CBC: Complete blood count

Parameter	Min	Max	Mean	SD
FEV1/FVC	0.34	0.69	0.6	0.08
FEV1%	21	97	57.94	22.42
MPV (fL)	6.8	12.3	9.37	1.46
MPV after 1 month (fL)	7.1	12.1	9.4	1.22
TLC	4.7	15.9	9.92	2.92
Platelet count	146	504	300.5	93.38

Table 3 indicates no significant correlation between MPV and age, BMI, or platelet count. However, a significant positive correlation was observed between the initial MPV measurement and both FEV1/FVC (r = 0.57) and FEV1% (r = 0.73). In contrast, a significant negative correlation was found with TLC (r = -0.536).

**Table 3:** Correlations of MPV among all studied cases BMI: Body mass index; FEV1: Forced expiratory volume at one second; FVC: Forced vital capacity; MPV: Mean platelet volume; TLC: Total leukocyte count p ≤ 0.05: significant (*); p > 0.05: insignificant

Parameter	r	p
Age	-0.19	0.1
BMI	-0.02	0.8
FEV1/FVC	0.57	0.00*
FEV1%	0.73	0.00*
TLC	-0.536	0.00*
Platelet count	0.09	0.4

Table 4 shows that age, gender, and BMI were statistically insignificantly different between the two groups.

**Table 4:** Comparison of sociodemographic data between the exacerbation and stable groups BMI: Body mass index

Variable		Excacerbation group (N = 34)	Stable group (N = 34)	t	p
Age (years)	Min-Max	42-70	41-70	1.274	0.207
	Mean ± SD	54.97 ± 7.87	52.58 ± 7.54		
Gender	Male, n (%)	27 (79.41%)	25 (73.52%)	0.326	0.567
	Female, n (%)	7 (20.58%)	9 (26.47%)		
BMI (kg/m^2^)	Min-Max	18.6-28.6	17.5-29.1	0.686	0.494
	Mean ± SD	22.65 ± 2.77	22.18 ± 2.87		

Table 5 shows that among the exacerbation group, FEV1/FVC ranged from 0.34 to 0.69, with a mean (± SD) of 0.53 (± 0.08), whereas among the stable group, FEV1/FVC ranged from 0.55 to 0.69, with a mean (± SD) of 0.66 (± 0.03). This was significantly higher among the stable group.

**Table 5:** Comparison of spirometry measures between the exacerbation and stable groups FEV1: Forced expiratory volume at one second; FVC: Forced vital capacity p ≤ 0.05: significant (*); p > 0.05: insignificant

Parameter		Excacerbation group (N = 34)	Stable group (N = 34)	t	p
FEV1/FVC	Min-Max	0.34-0.69	0.55-0.69	-7.95	0.001*
	Mean ± SD	0.53 ± 0.08	0.66 ± 0.03		
FEV1%	Min-Max	21-57	65-97	-17.28	0.001*
	Mean ± SD	37.79 ± 11.14	78.08 ± 7.79		

**Figure 1:**
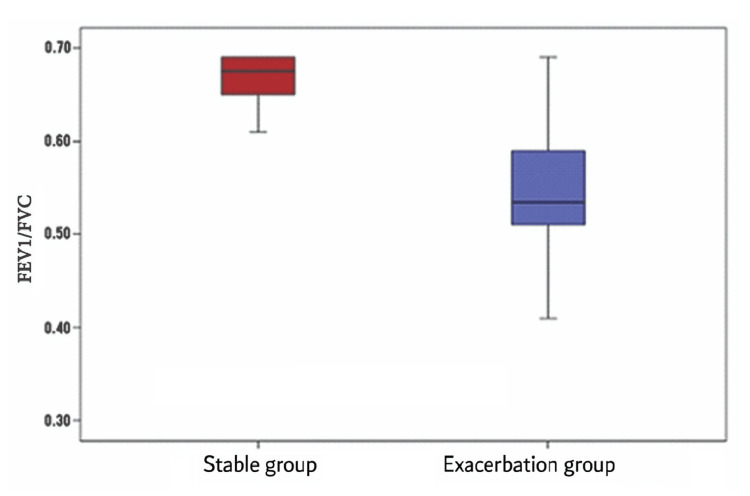
FEV1/FVC difference between the exacerbation and stable groups The x-axis represents two clinical groups of patients: the stable COPD group and the exacerbation group. The y-axis represents the FEV1/FVC ratio. FEV1: Forced expiratory volume at one second; FVC: Forced vital capacity; COPD: Chronic obstructive pulmonary disease

Among the exacerbation group, FEV1% ranged from 21 to 57, with a mean (± SD) of 37.79 (± 11.14), whereas among the stable group, FEV1% ranged from 65 to 97, with a mean (± SD) of 78.08 (± 7.79). This was significantly higher among the stable group (Figure 1).

Table 6 shows that there were significant differences between the exacerbation group and the stable group regarding initial MPV, follow-up MPV, TLC, and platelet count.

**Table 6:** Comparison of CBC parameters between the exacerbation and stable groups MPV: Mean platelet volume; TLC: Total leukocyte count; CBC: Complete blood count p ≤ 0.05: significant (*); p > 0.05: insignificant

Parameter		Excacerbation group (N = 34)	Stable group (N = 34)	t	p
MPV (fL)	Min-Max	6.8-10.2	8.1-12.3	-7.55	0.001*
	Mean ± SD	8.37 ± 1.04	10.36 ± 1.12		
MPV after 1 month (fL)	Min-Max	7.1-10.9	8.4-12.1	-5.62	0.001*
	Mean ± SD	8.71 ± 1	10.09 ± 1.12		
TLC	Min-Max	9.3-15.9	4.7-11.1	10.96	0.00*
	Mean ± SD	12.25 ± 1.55	7.59 ± 1.92		
Platelet count	Min-Max	190-504	146-428	2.57	0.01*
	Mean ± SD	328.02 ± 99.3	272.97 ± 88.75		

**Figure 2:**
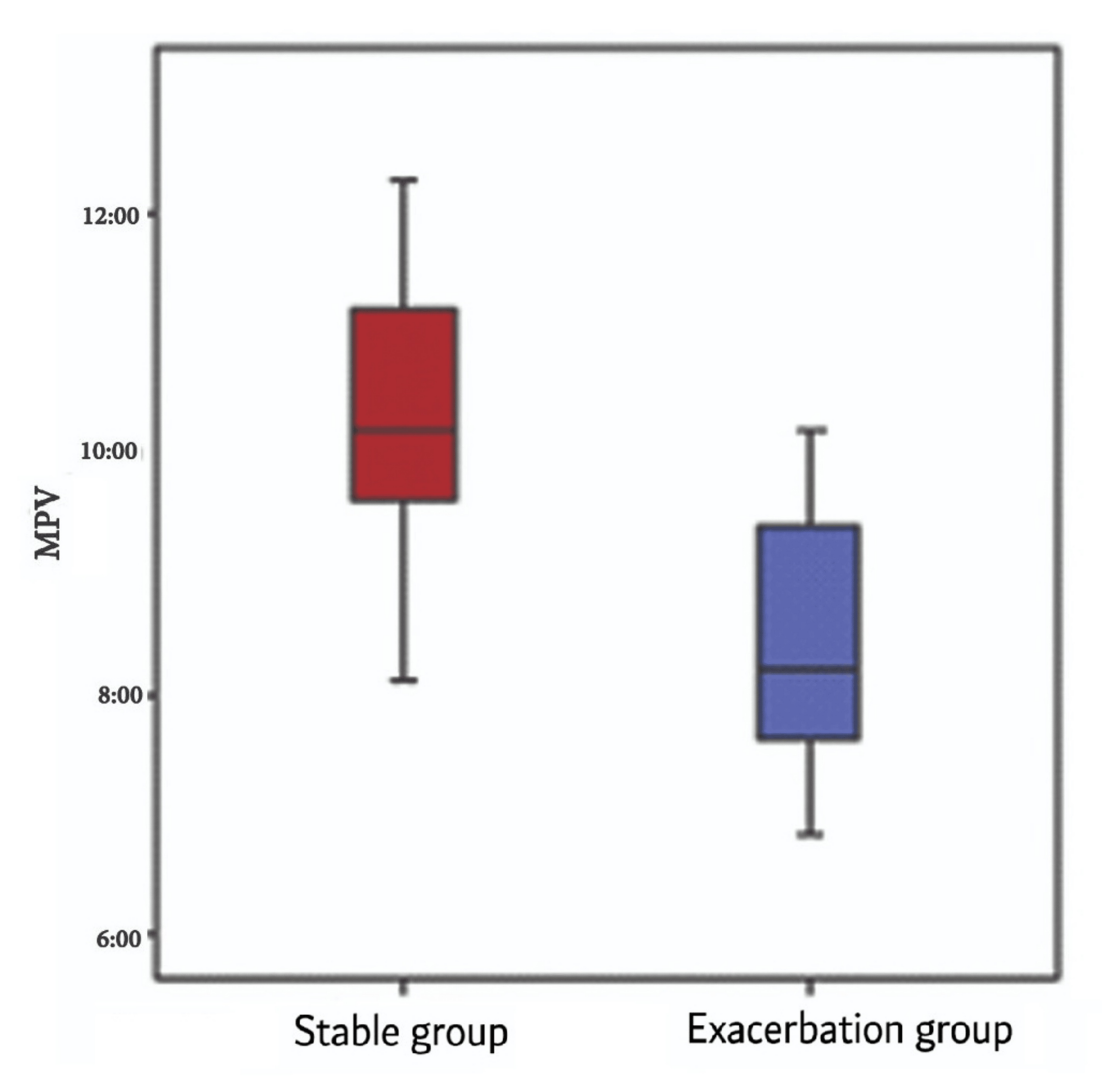
MPV difference between the exacerbation and stable groups The x-axis represents two clinical groups of patients: the stable COPD group and the exacerbation group. The y-axis represents the MPV. MPV: Mean platelet volume; COPD: Chronic obstructive pulmonary disease

The initial and follow-up MPV levels were significantly higher in the stable group (mean ± SD: 10.36 ± 1.12 vs. 8.37 ± 1.04, respectively) compared to the exacerbation group (mean ± SD: 10.09 ± 1.12 vs. 8.71 ± 1, respectively) (Figure 2).

**Figure 3:**
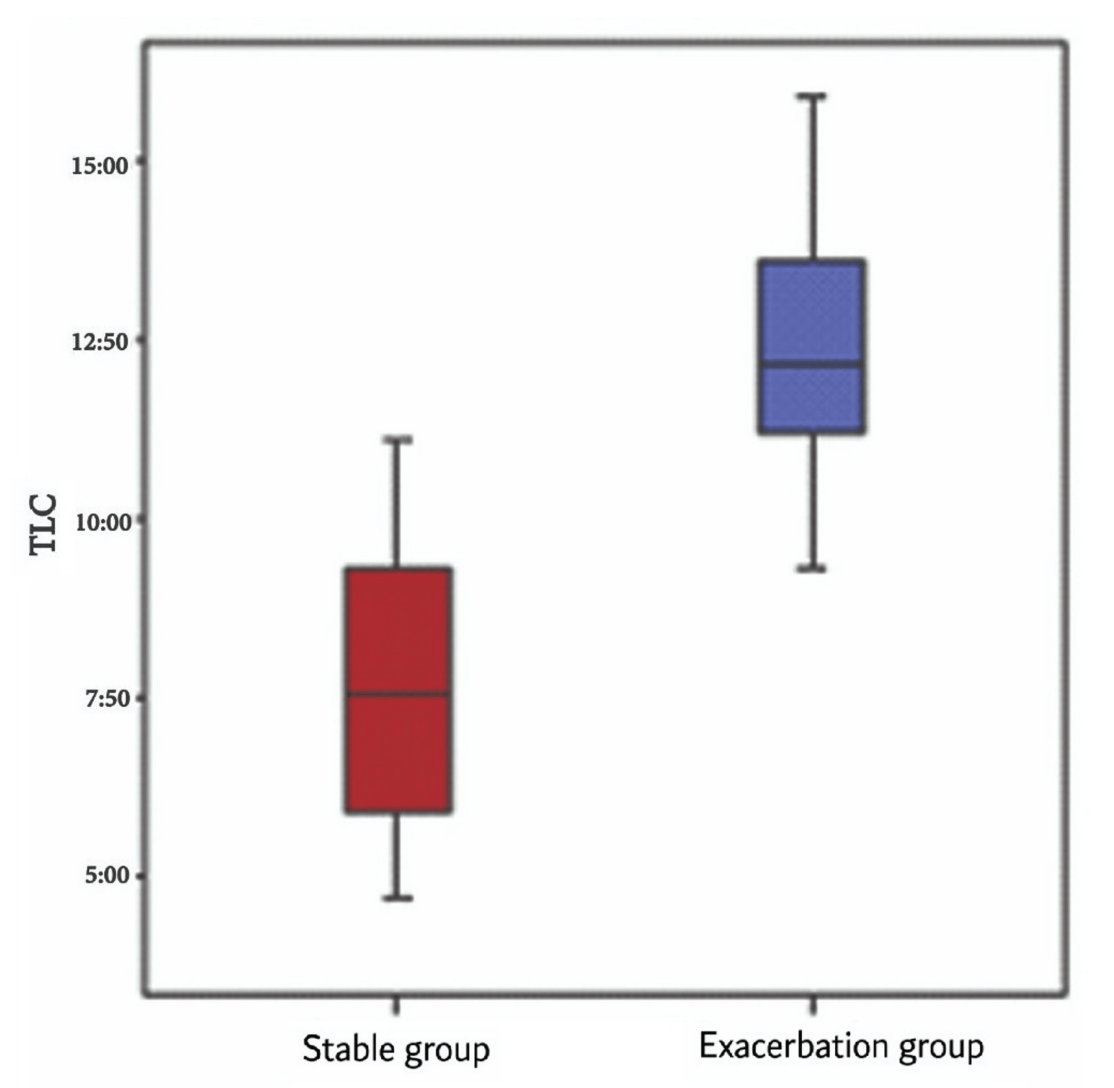
TLC difference between the exacerbation and stable groups The x-axis represents two clinical groups of patients: the stable COPD group and the exacerbation group. The y-axis represents the TLC. TLC: Total leukocyte count; COPD: Chronic obstructive pulmonary disease

Conversely, TLC and platelet count were significantly elevated in the exacerbation group (mean ± SD: 12.25 ± 1.55 vs. 7.59 ± 1.92, respectively) compared to the stable group (328.02 ± 99.3 vs. 272.97 ± 88.75, respectively) (Figure 3).

Exacerbation group

**Figure 4:**
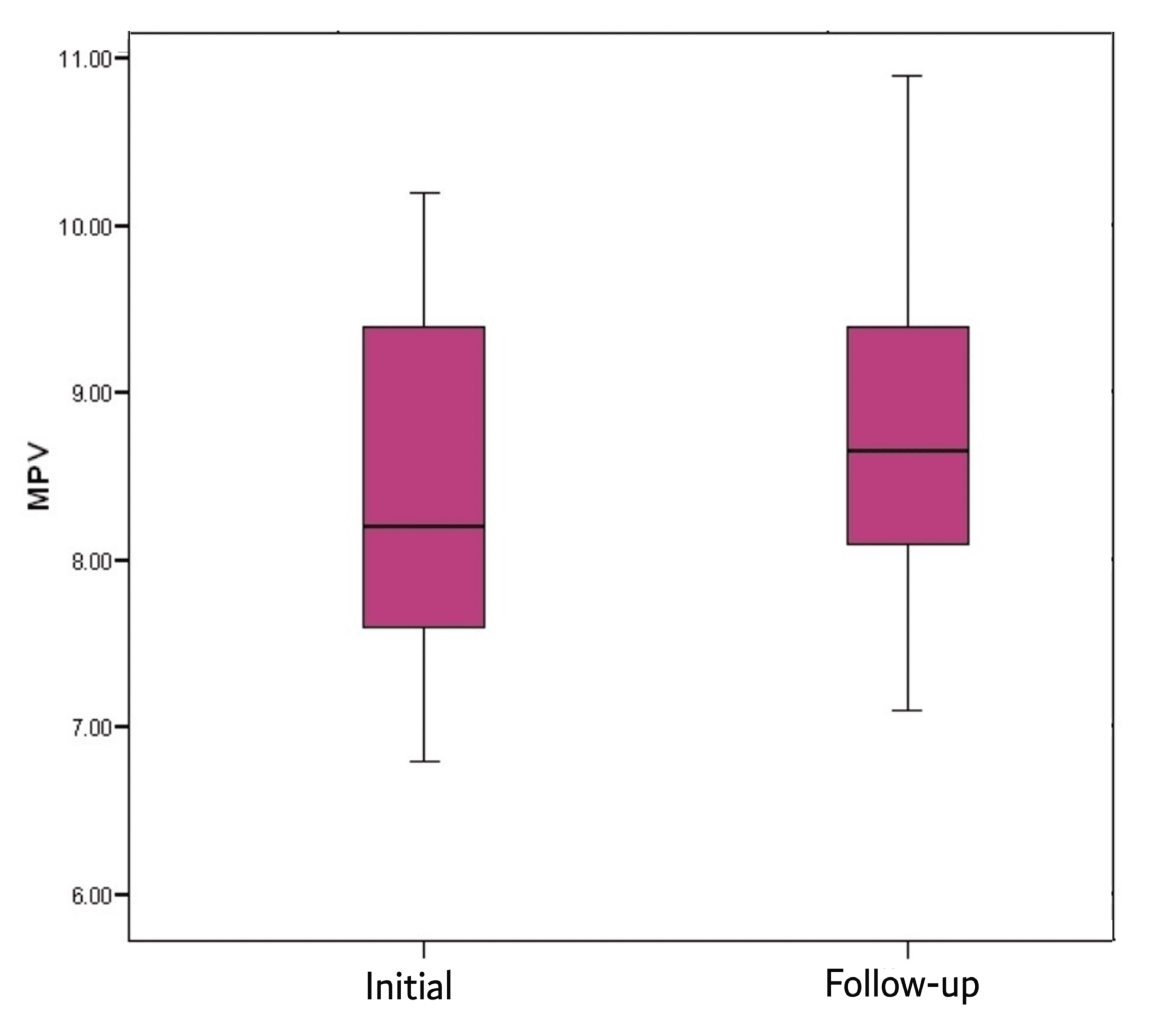
Initial and follow-up MPV in the exacerbation group The x-axis represents the two time points when the MPV values were noted. The y-axis represents the MPV. MPV: Mean platelet volume

Table 7 shows that among the exacerbation group, the mean initial MPV was 8.37 ± 1.04 fL, and the mean follow-up MPV was 8.71 ± 1 fL, with a statistically significant difference. MPV significantly increased after one month of treatment (Figure 4).

**Table 7:** Comparison between initial MPV and MPV after one month in the exacerbation group MPV: Mean platelet volume p ≤ 0.05: significant (*); p > 0.05: insignificant

Parameter	Mean	SD	t	p
MPV (fL)	8.37	1.04	-3.61	0.001*
Follow-up MPV (fL)	8.71	1		

Table 8 indicates that the associations between MPV and age, BMI, FEV1/FVC, FEV1%, and platelet count were not statistically significant. However, a significant positive correlation was observed between initial MPV and follow-up MPV. Conversely, in individuals experiencing an exacerbation, MPV showed a significant negative correlation with TLC. 

**Table 8:** Correlations of MPV within the exacerbation group BMI: Body mass index; FEV1: Forced expiratory volume at one second; FVC: Forced vital capacity; MPV: Mean platelet volume; TLC: Total leukocyte count p ≤ 0.05: significant (*); p > 0.05: insignificant

Parameter	r	p
Age	-0.2	0.2
BMI	-0.12	0.4
FEV1/FVC	0.19	0.2
FEV1	0.33	0.056
MPV after 1 month	0.85	0.00*
TLC	-0.58	0.00*
Platelet count	0.16	0.3

Stable group

Table 9 shows a significant difference between initial MPV and follow-up MPV (mean ± SD: 10.36 ± 1.12 vs. 10.09 ± 1.01, respectively).

**Table 9:** MPV before and after one-month follow-up in the stable group MPV: Mean platelet volume p ≤ 0.05: significant (*); p > 0.05: insignificant

Parameter	Mean	SD	t	p
MPV (fL)	10.36	1.12	3.52	0.001*
Follow-up MPV (fL)	10.09	1.01		

## Discussion

Globally, COPD is a common cause of mortality and morbidity and is primarily characterized by restriction of ventilation, which is the consequence of inflammation and airway remodeling. Increasing evidence indicates that COPD is a multifaceted condition that involves more than just the obstruction of airflow [13].

All cases

Findings from our study indicated that there were no significant correlations between MPV and any of the following factors: age, BMI, or platelet count. Conversely, we found a strong direct correlation between the first measurement of MPV and both FEV1/FVC and FEV1% (r = 0.57 and 0.73, respectively). Alongside this, there was also a significant indirect correlation with TLC (r = -0.536).

Similarly, Helmy et al. conducted a study on 135 adult patients. The patients were divided into three equal groups: Group I had acute exacerbation of COPD, Group II consisted of healthy smokers without COPD, and Group III included healthy controls. They demonstrated a substantial positive correlation between FEV1 and MPV in the entire sample (r = 0.449, p ≤ 0.001) [14]. In contrast, Cui et al. recruited 116 very old male outpatients with COPD and observed a negative correlation between FEV1% predicted and MPV (b = -0.384, p = 0.0001) [3]. The variance may be due to different inclusion criteria.

According to our findings, FEV1/FVC and FEV1% were significantly higher among the stable group than the exacerbation group. In agreement with our study, Rashed et al. conducted a study on 100 patients with acute exacerbation of COPD and 100 patients with stable COPD matched for age and gender. They found a significant decrease in FEV1/FVC and FEV1% in the acute exacerbation group compared to the stable group [15].

In the current study, there were significant differences between the exacerbation group and the stable group with respect to initial MPV and follow-up MPV; these were significantly higher among the stable group in comparison to the exacerbation group. Among the exacerbation group, the mean initial MPV was 8.37 ± 1.04 fL and the mean follow-up MPV was 8.71 ± 1 fL, with the difference being statistically significant. Parallel to our study, Ali et al. included a total of 80 COPD patients divided equally into stable and exacerbated groups, revealing a significant decrease in MPV within the exacerbated patient group compared to the stable one [16].

Additionally, Wang et al. investigated 70 patients with an acute exacerbation of COPD at admission and reassessed them when stable, alongside 70 healthy controls. Their study found that participants with an exacerbation of COPD had lower MPV compared to patients in the stable phase of COPD and healthy controls [17].

Also, Şahin et al. included three groups: stable COPD (Group 1, n = 140), COPD with acute exacerbation (Group 2, n = 110), and healthy controls (Group 3, n = 50). MPV values were significantly lower in patients with acute exacerbation of COPD compared to patients with stable COPD (p < 0.05) [18].

In our study, there were significant differences between the exacerbation group and the stable group regarding TLC; TLC was significantly higher among the exacerbation group compared to the stable group. In line with our study, Şahin et al. investigated TLC values and found that they were significantly higher in patients with acute exacerbation of COPD compared to patients with stable COPD [18].

Exacerbation group

Based on our study, there were insignificant correlations between MPV and age, BMI, FEV1/FVC, FEV1%, and platelet count among the exacerbation cases. Meanwhile, there was a significant direct correlation between initial and follow-up MPV. In contrast, there was a significant indirect correlation between TLC and MPV among the exacerbation cases.

These results are in agreement with Ma et al., who found an insignificant relationship between MPV and FEV1% in the COPD exacerbation cases [19]. Nevertheless, Eltaweel et al. enrolled 25 stable and 25 exacerbation COPD patients selected in a non-randomized manner and found a statistically significant positive connection between MPV and spirometric parameters FEV1% and FEV1/FVC ratio in acute exacerbation of COPD patients [20].

In the current study, there was a significant difference between initial MPV and follow-up MPV (mean ± SD: 8.37 ± 1.04 vs. 8.71 ± 1, respectively). These results nearly coincide with Ali et al., who found a significant increase in MPV (mean ± SD: 9.9 ± 0.4) during the stable state in follow-up visits after two weeks compared to during exacerbations (mean ± SD: 8.7 ± 0.9) [16].

Limitations

Being a single-center study with a small sample size, the generalizability of the findings may be limited. Additionally, we did not classify patients according to severity. Our study excluded COPD patients with comorbidities and those receiving medical treatment.

## Conclusions

MPV serves as a rapid and reliable indicator of inflammatory response. Additionally, it may serve as a dependable biomarker for evaluating the inflammatory response during exacerbations of COPD. MPV was found to be decreased in acute exacerbations of COPD compared to smokers and healthy controls. Evaluation of MPV in COPD exacerbation may indicate systemic inflammation. Thus, MPV may be used as a negative acute-phase reactant in COPD exacerbations.
